# Malaria Impairs T Cell Clustering and Immune Priming despite Normal Signal 1 from Dendritic Cells

**DOI:** 10.1371/journal.ppat.0030143

**Published:** 2007-10-12

**Authors:** Owain R Millington, Vivienne B Gibson, Catherine M Rush, Bernd H Zinselmeyer, R. Stephen Phillips, Paul Garside, James M Brewer

**Affiliations:** 1 Centre for Biophotonics, University of Strathclyde, Glasgow, United Kingdom; 2 Division of Infection and Immunity, Glasgow Biomedical Research Centre, University of Glasgow, Glasgow, United Kingdom; University of Oxford, United Kingdom

## Abstract

Interactions between antigen-presenting dendritic cells (DCs) and T cells are essential for the induction of an immune response. However, during malaria infection, DC function is compromised and immune responses against parasite and heterologous antigens are reduced. Here, we demonstrate that malaria infection or the parasite pigment hemozoin inhibits T cell and DC interactions both in vitro and in vivo, while signal 1 intensity remains unaltered. This altered cellular behaviour is associated with the suppression of DC costimulatory activity and functional T cell responses, potentially explaining why immunity is reduced during malaria infection.

## Author Summary

Malaria is a major infectious disease, affecting 500 million people and causing 2.7 million deaths each year. The severity of malaria is, in part, due to the failure of the host immune system to effectively clear an infection and generate protective immunity. Dendritic cells (DCs) are central to the immune system; by presenting components of pathogens to circulating T cells, they are able to initiate a highly specific immune response to clear an infection. Importantly, the quality of the interaction between T cell and DCs can affect the functional outcome of the immune response. However, previous work has demonstrated that DCs are modified by malaria parasites, resulting in inefficient priming of the adaptive immune system. Here, we have visualised the interactions between DCs and T cells in the context of malaria and demonstrate that infection is able to prevent priming of immune responses by antagonising these cell–cell contacts. Importantly, the failure to form long-lasting interactions is not due to reduced presentation of antigens by the DC, suggesting that other mechanisms may be involved. These studies provide a visual insight into the mechanism by which parasites may suppress immunity and highlight the importance of early cellular interactions in the immune response.

## Introduction

The activation of a protective and highly specific immune response requires a system that can survey, decipher, and quickly respond to infection in an appropriate manner. Dendritic cells (DCs) participate in all of these important activities, and upon detection of a “danger signal”, rapidly mature and express molecules required to generate the antigen-specific (signal 1) and costimulatory signals (signal 2) required to induce the activation of antigen-specific CD4+ “helper” T cells [1]. These cellular interactions are an essential step in initiation of adaptive immune responses, and factors that influence signal 1 and 2 upon DCs, such as the dose and duration of antigen presentation [2,3] and the level of activation of the DC [4], can all affect the type of adaptive immune response induced. Importantly, differences in the behaviour of T cells can be observed in vivo by comparing situations in which T cells are primed (i.e., activated to induce a protective immune response) or tolerised/regulated (i.e., where they become functionally hyporesponsive). Thus, during tolerance induction, clusters of T cells are smaller compared with priming responses [5], and regulatory T cells (Tregs) are known to prevent stable T cell–DC interactions [6,7].

Malaria represents a global health challenge, with approximately 500 million clinical cases reported annually [8]. *Plasmodium* can induce immunosuppression in infected individuals, resulting in an increased susceptibility to secondary infections and reduced vaccine efficacy in patients and in animal models [9–14]. Suppression of immune responses is in part associated with the uptake of the malaria pigment hemozoin (HZ) [15,16]. Although certain studies have suggested that HZ activates DCs [17,18], others have demonstrated that DCs are functionally less responsive during malaria infection [19], and that HZ is able to regulate DC activation [20,21]. We have recently shown that the modulation of DCs by malaria infection and/or HZ significantly reduces T cell expansion, cytokine production, and migration into B cell follicles [21], explaining the immunosuppression observed in infected individuals [9–14]. Importantly, the modulation of DC function (and consequently immunity) is a highly dynamic phenomenon, which changes throughout the course of infection, with immune suppression most evident soon after the peak of infection. This affects immune responsiveness, with T cell and B cell responses to heterologous antigens altered kinetically during the course of malaria infection [21].We were therefore interested in examining whether the modulation of interactions between DCs and T cells by malaria infection could account for this reduced immunity. Using in vitro and in vivo systems to examine the behaviour of these cells, we have demonstrated that the immune suppression during malaria infection is associated with impaired DC–T cell interactions.

## Results/Discussion

**Figure 1:**
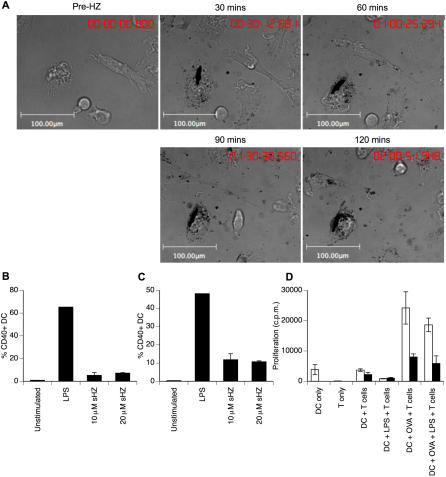
HZ Uptake by DCs Reduces Effector Function (A) BM-derived DCs were visualised by light microscopy prior to and following the addition of 40 μM HZ. Images show the same field of view at 30-min intervals (full video available as Video S1). (B) BM DCs were cultured with the indicated doses of HZ, or with 1 μg/ml LPS. After 24 h, the proportion of CD11c+ DCs expressing CD40 was determined by flow cytometry. (C) After 24 h culture with HZ, 1 μg/ml LPS was added to DCs and the proportion of cells expressing CD40 was analysed 18 h later. (D) DCs were treated with HZ as above (filled bars) or remained untreated (empty bars). DCs were subsequently pulsed with OVA (1 mg/ml) for 6 h prior to stimulation with LPS for 18 h as indicated. DCs were then mixed at a 1:1 ratio with OVA-specific DO11.10 T cells and proliferation assessed by [^3^H] thymidine incorporation for the last 18 h of a 72 hour culture. Results show the mean of triplicate samples ± 1 s.d. sHZ, synthetic HZ.

To dissect the effects of the malaria pigment on DCs, we first examined the uptake of HZ over time as well as DC activation, as assessed by increased expression of the costimulatory molecule, CD40 (Figure 1). Bone marrow (BM)–derived DCs readily phagocytosed HZ in vitro, with pigment taken up within 5 min of addition and accumulating steadily in the majority of DCs over the following 2 h (Figure 1A and Video S1). Addition of synthetic HZ [22] to DCs not only failed to induce upregulation of CD40 (Figure 1B), but also reduced the subsequent responsiveness of DCs to lipopolysaccharide (LPS) stimulation (Figure 1C), confirming our previous observations with HZ isolated from P. falciparum (Figure S1 and [21]). Furthermore, the morphological changes typically associated with LPS-induced activation of DCs were not observed in HZ-treated DCs by time-lapse microscopy (Videos S2 and S3). The central function of DCs is presentation of antigen to CD4+ T cells in order to initiate the adaptive immune response. We therefore assessed the ability of HZ-loaded DCs to stimulate ovalbumin (OVA)-specific CD4+ T cell proliferation. As expected, OVA-specific DO11.10 T cells proliferated following incubation with OVA-pulsed DCs or LPS-stimulated OVA-pulsed DCs. This proliferation was significantly reduced if the DCs were treated with HZ prior to addition of antigen (Figure 1D), as was subsequent T cell cytokine production (unpublished data). Thus, DCs exposed to HZ rapidly accumulate pigment, rendering them functionally impaired with reduced LPS responsiveness and a failure to induce efficient T cell responses.

**Figure 2:**
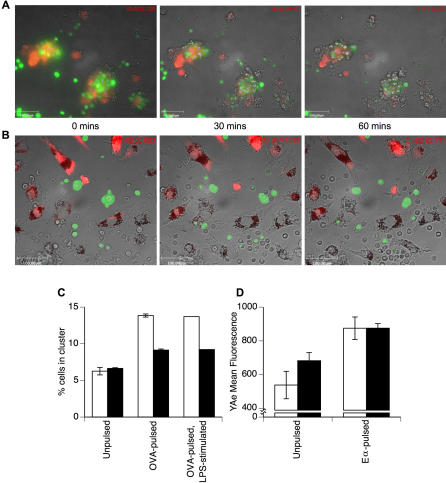
In Vitro DC–T Cell Interactions Are Inhibited by HZ (A and B) BM DCs remained untreated (A) or were treated with HZ (B) prior to pulse with OVA, as described in Figure 1. DCs were then fluorescently labelled with CMRA (orange) and mixed at a 1:1 ratio with CFSE-labelled DO11.10 T cells (green). Cellular interactions were imaged 20 h after mixing by bright field and fluorescence microscopy. Images show the same field of view at 30-min intervals (full sequences available as Videos S4 and S5). (C) DCs and T cells were treated, fluorescently labelled, and co-cultured as described above. Twenty hours after mixing, cells were fixed in situ by adding 4% paraformaldehyde. Cells were then recovered and analysed by flow cytometry to determine the proportion of CMRA+ CFSE+ conjugates in cultures containing untreated (empty bars) or HZ-treated DCs (filled bars). Results show the mean ± 1 s.d. of triplicate samples and are representative of three similar experiments. (D) Untreated (empty bars) or HZ-treated (filled bars) DCs were pulsed with 100 μg Eα-GFP and antigen presentation by CD11c+ cells assessed 6 h later by flow cytometry. Results show the mean fluorescence ± 1 s.d. of triplicate samples and are representative of two similar experiments.

The initial interactions between DC and T cell have important implications for the functional outcome of the T cell response [2–4]. Since we had observed reduced T cell responses following stimulation with HZ-treated DCs, we next examined in vitro the interactions between T cells and DCs that are involved in the induction of an antigen-specific response. DCs were labelled with the fluorescent dye CMRA (orange) and pulsed with OVA before mixing with CFSE-labelled (green) OVA-specific CD4+ T cells. Twenty hours later, T cells were observed clustered around DCs, with several CFSE+ T cells remaining closely associated with CMRA+ DCs for up to 2 h (Figure 2A and Video S4). However, in cultures in which the DCs had been treated with HZ prior to antigen pulse, these clusters were not evident, with CFSE-labelled T cells making transient contacts with CMRA+ DCs lasting only a few minutes (Figure 2B and Video S5). To quantify this clustering of T cells around DCs, we employed an assay in which fluorescently labelled DCs and T cells were co-cultured as described above and then fixed using paraformaldehyde prior to analysis by flow cytometry. In this way, CMRA+ DCs, CFSE+ OVA-specific T cells, and clusters of DCs and T cells could be individually identified (Figure S2). Addition of antigen to this system increased the proportion of clustered cells (detected as CMRA+ CFSE+; Figure 2C). However, this colocalisation of T cells with DCs was reduced in cultures in which the DCs were pre-treated with HZ (Figure 2C), confirming the above observations.

The above data suggest that HZ-treated DCs are altered such that their ability to interact with naïve CD4+ T cells is suppressed, resulting in reduced T cell proliferation. One possible explanation for this observation is that HZ-loaded DCs are simply unable to take up, process, or present antigen. Use of GFP-labelled Eα antigen [23] allowed us to measure antigen uptake by DCs, as well as antigen presentation using the YAe antibody that specifically recognises the complex of Eα-peptide in the context of class II major histocompatibility complex (MHC) [24]. In this system, DCs pre-treated with HZ were able to process and present antigen as effectively as untreated DCs (Figure 2D). Importantly, DCs co-cultured with *P. chabaudi–*infected erythrocytes (that produce HZ deposition within DCs and recapitulate the described effects on T cells [21]) also presented Eα-peptide to the same extent as resting DCs or DCs cultured with uninfected erythrocytes (Figure S3). Thus, whilst HZ-treated DCs are able to process antigen efficiently and provide a “signal 1” in the form of antigen/MHC for the T cell, their interaction with CD4+ T cells is altered such that stable clusters do not form to initiate a functional T cell response.

**Figure 3:**
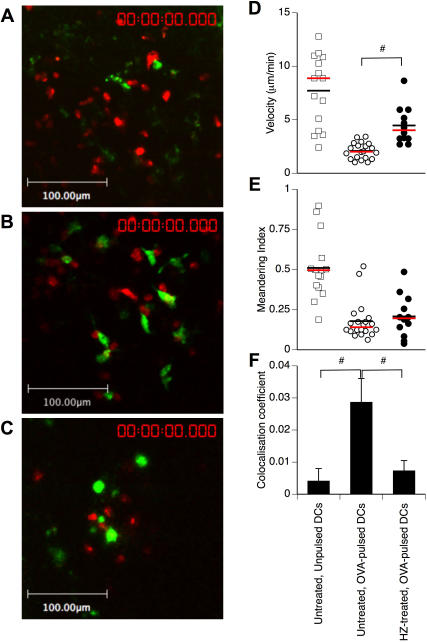
DC–T Cell Interactions Are Inhibited In Vivo by HZ BALB/c mice were injected with CMTPX-labelled DO11.10 T cells (red) and CFSE-labelled DCs (green) which had been untreated (A), pulsed with OVA_323–339_ (B), or treated with HZ prior to OVA_323–339_ pulse (C). Cellular interactions in the draining popliteal LNs were imaged by multiphoton microscopy 24 h later. Images represent a z-compressed 30-μm stack from a single time point (full sequences available as Videos S6–S8). T cell migration was analysed using Volocity software (Improvision) and the mean velocity (D) and meandering index (E) of cells calculated. Mean and median values are represented by the red and black lines, respectively. DC–T cell interaction was measured by quantifying the colocalisation of green voxels with red to determine the proportion of DC volume in contact with T cells (F). Data shown are representative of three similar experiments (# *p* < 0.05).

In order to confirm the above in vitro observations in vivo, we used multi-photon laser scanning microscopy to examine the interactions between DCs and T cells in intact lymph nodes (LNs). BALB/c mice received CMTPX-labelled DO11.10 T cells and were subsequently immunised subcutaneously with CFSE-labelled DCs. Twenty-four hours after immunisation with untreated DCs, a small proportion of these cells had migrated to the draining LN and could be seen making transient contacts with OVA-specific CD4+ T cells (Figure 3A and Video S6), as previously described [25–27]. When DCs were pulsed with OVA prior to transfer, more interactions between DCs and antigen-specific T cells were observed in the draining LN, and these contacts appeared to last longer (Figure 3B and Video S7). While interactions between antigen-specific T cells and DCs were also observed following injection of OVA-pulsed, HZ-treated DCs, these appeared more motile, and less stable, than the clustering seen with normal antigen-pulsed DCs (Figure 3C and Video S8). The 4-dimensional movement of the OVA-specific T cells was characterised by software-based tracking. As previously described [28], the 3-dimensional velocity of antigen-specific CD4+ T cells was reduced by pulsing DCs with OVA prior to transfer as T cells clustered around DCs (Figure 3D–3F). However, T cells stimulated by HZ-treated, OVA-pulsed DCs had higher mean velocities than those of T cells undergoing an effective priming response, although this was also significantly slower than naïve CD4+ T cells (Figure 3D). Despite this difference in velocity, the meandering index (a measure of directionality of cell movement) of T cells in recipients of normal OVA-pulsed or HZ-treated OVA-pulsed DCs was reduced to the same extent compared with naïve T cells (Figure 3E). Furthermore, responding T cells upregulated the early activation marker CD69 in recipients of OVA-pulsed DCs, irrespective of whether they were loaded with HZ or not (unpublished data), demonstrating that T cells were recognising antigen presented by the DCs. In order to quantify the interactions between DCs and T cells, we analysed the degree of colocalisation between green DCs and red T cells to calculate a colocalisation coefficient representing the proportion of green voxels that were also red. Whilst T cell interaction with normal DCs was increased following antigen recognition, the colocalisation of DCs and T cells was inhibited by HZ treatment of DCs (Figure 3F). Thus, it appears that the in vivo interactions between CD4+ T cells and HZ-loaded DCs are altered relative to a priming response, despite recognition of antigen by T cells, confirming our in vitro observations.

**Figure 4:**
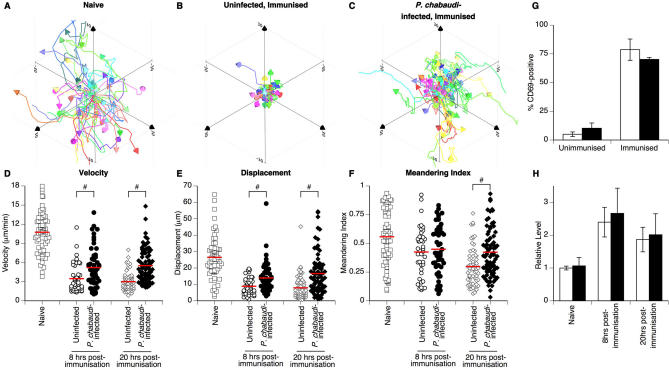
Altered T Cell Motility during Malaria Infection BALB/c mice were infected with 10^6^ P. chabaudi AS-infected erythrocytes i.p. Twelve days later, infected mice and uninfected groups received CFSE-labelled DO11.10 T cells and were challenged with OVA/LPS intravenously (B and C). Control animals remained unimmunised (A). T cell motility in LNs was imaged by multiphoton microscopy 8 and 20 h later. T cell behaviour was analysed using Volocity software and the tracks of several individual cells plotted (A–C) (full sequences available as Videos S9–S11). The mean velocity (D), displacement (E), and meandering index (F) of cells from multiple samples from multiple mice were also calculated. Mean and median values are represented by the red and black lines, respectively. Expression of CD69 on OVA-specific CD4+ T cells was assessed 20 h after immunisation of uninfected (empty bars) or P. chabaudi–infected (filled bars) animals (G). Antigen presentation was assessed following immunisation of uninfected (empty bars) or P. chabaudi–infected (filled bars) C57BL/6 mice with 500 μg Eα-GFP + 50 ng LPS intravenously YAe staining on CD11c+ splenic DCs was assessed 8 and 20 h after immunisation. Data shown are representative of three similar experiments (# *p* < 0.05 uninfected, OVA-immunised versus P. chabaudi–infected, OVA-immunised).

We next wanted to confirm the importance of the above observations during malaria infection. We have previously demonstrated that around day 12 after infection with P. chabaudi, OVA-specific CD4+ T cells fail to proliferate effectively to challenge with antigen and that as a consequence, T cell migration into B cell follicles is suppressed and antibody responses are not induced [21]. We therefore characterised the migratory behaviour of OVA-specific CD4+ T cells following immunisation of malaria-infected animals (Figure 4A–4C and Videos S9–S11). When the movement of multiple cells in several samples was analysed, it was evident that these T cells moved less rapidly and migrated shorter distances 8 and 20 h following immunisation, compared with naïve T cells (Figure 4D–4F). Although T cells transferred into malaria-infected animals immunised with OVA moved more slowly than naïve T cells, their velocities were significantly higher than those of primed T cells in uninfected recipients at both time points following immunisation (Figure 4D). T cells activated in P. chabaudi–infected animals also moved greater distances away from their point of origin than T cells activated in uninfected animals (Figure 4E and 4F). These differences were observed at a time when lymphoid architecture was essentially normal [21] and no differences were apparent between the behaviour of naïve T cells in uninfected and P. chabaudi–infected animals (Figure S4). Despite this difference in behaviour, OVA-specific T cells upregulated the early activation marker CD69 in response to immunisation in both uninfected and P. chabaudi–infected animals (Figure 4G), suggesting that antigen is presented effectively to T cells during malaria infection. In order to confirm that antigen presentation was unaltered by malaria infection, P. chabaudi–infected C57BL/6 mice were immunised with 500 μg of Eα-GFP plus 50 ng of LPS on day 12 of infection. Eight or 20 h later, splenic DCs isolated from malaria-infected animals showed similar surface levels of peptide/MHC (signal 1) as DCs from uninfected animals (Figure 4H). Therefore, the lack of formation of long-lasting interactions between DCs and T cells that is associated with reduced effector functions of T cells in malaria-infected animals is signal 1 independent.

Several reports have demonstrated immunosuppression during malaria infection in patients and animal models [9–14]. We [21] and others [20] have shown that DC activation and function is suppressed by infection or malaria pigment and, as a consequence, T cell and B cell responses fail to develop effectively. Here we have shown that uptake of HZ by DCs modulates their ability to interact effectively with T cells, despite presenting normal levels of peptide/MHC on their surface. The failure of T cells to fully interact with DCs appears to result in a lack of efficient T cell priming, leading to the subsequent failure of a protective immune response. Thus, these observations may explain why T cell responses fail to develop during malaria infection and clearly demonstrates the significance of these early interactions in the generation of an immune response.

It is important to highlight that the impact of malaria on the ability of the immune system to respond changes markedly during the different phases of the infection. Thus, DC function is impaired immediately following the initial burst of parasitaemia. As a consequence, T cell proliferation, effector function, and migration are suppressed, and thus B cells do not receive help for expansion or antibody production [21]. We were interested in examining why T cell responses fail at this important time point and reasoned that the interactions between DCs and T cells might play an important role. By analysing the behaviour of T cells in malaria-infected animals following the peak of parasitaemia (a period of immune suppression), we demonstrated that the failure of immunity is associated with reduced interaction between T cells and endogenous antigen-presenting cells (Figure 4). Such immune suppression is not as evident at earlier and later time points of infection, but it will be interesting to examine how DC–T cell interactions are affected at these stages. Importantly, transfer of antigen-pulsed, HZ-treated DC into naïve, uninfected recipients recapitulated these observations, suggesting that while pro-inflammatory infections such as malaria may be followed by a wave of anti-inflammatory cytokine production [29], failure of T cells to interact with DC effectively is sufficient to suppress immune priming.

In this investigation, we confirmed several previous reports that DCs pulsed with HZ displayed an impaired ability to undergo subsequent activation. It is important to note that others have suggested a pro-inflammatory role for HZ, possibly via the pathogen recognition receptor TLR9 [17]. Although, in our hands, DCs treated with HZ show a minor upregulation of CD40, this is significantly lower than the activation seen by stimulating DCs with LPS (Figure 1B), and these HZ-treated DCs fail to respond to subsequent activation (Figure 1C). It is possible that these differences reflect the different subsets of DCs used, since TLR9 is primarily expressed by Flt3L-generated, plasmacytoid-like DCs [30], as opposed to the granulocyte/monocyte colony stimulating factor (GM-CSF)-induced, predominantly myeloid DCs used here. It is also possible that the observed differences reflect the methods used to produce synthetic HZ from β-hematin (unpublished data). Recent work has demonstrated the TLR9/MyD88-dependent activation of DC by parasite-derived HZ to be due to the binding of malarial nucleic acids to HZ [31]. In the current study we therefore used synthetic HZ. Importantly, synthetic HZ produced by our method recapitulates the effect upon DC function of both HZ isolated from parasites as well as the effect of *Plasmodium* infection in vivo (Figure S1 and [21]).

One explanation for our findings that HZ-loaded DCs are unable to form stable interactions with T cells is that the DCs express fewer costimulatory or adhesion molecules on their surface. Indeed, despite normal signal 1, HZ-treated DCs fail to upregulate costimulatory molecules in response to both TLR ligands and CD40 ligation (Figure 1 and [21]). Thus, although T cells are initially activated through antigen/MHC (achieving sufficient signal to express CD69), other molecules involved in synapse formation or T cell activation are not expressed by HZ-loaded DCs and stable clustering does not occur. This shows that signal 1 alone is not sufficient to drive T cell clustering and other factors, most likely costimulatory or adhesion molecules, are required for stable interactions between T cell and DC. Alternatively, active suppression of the T cell response may occur by the involvement of Tregs, which have been implicated in malaria infection [32]. Recent evidence has demonstrated that Tregs are able to suppress T cell activation by reducing their interactions with DCs in vivo [6,7], and this may be a possible mechanism for immune suppression during malaria infection.

Our results demonstrate the significance of the early interactions between T cells and DCs in the priming of effector T cell responses. Whilst the T cell–DC clustering dynamics are altered by HZ, DCs still present antigen to T cells, as demonstrated directly by YAe antibody staining and by the upregulation of CD69 by T cells. Thus, it is an inability to form stable, long-lasting clusters with HZ-laden DCs, independently of signal 1, that results in the reduced effector function of CD4+ T cells seen during malaria infection.

## Materials and Methods

### Animals and challenge infections.

Female BALB/c and C57BL/6 mice were purchased from Harlan Olac. The DO11.10 transgenic mice, with CD4+ T cells specific for OVA_323–339_ peptide in the context of I-A^d^ recognised by the KJ1.26 clonotypic antibody [33], were obtained originally from N. Lycke, University of Göteborg, Sweden, and backcrossed onto the scid background such that all lymphocytes were OVA-specific CD4+ T cells. All mice were maintained at the Biological Procedures Unit, University of Strathclyde, under specific pathogen-free conditions and first used between 6 and 8 wk of age in accordance with local and UK Home Office regulations.

To initiate a malaria infection, mice were inoculated with 1 × 10^6^ P. chabaudi AS-infected erythrocytes intraperitoneally. Parasitaemia was monitored by thin blood smears stained with Giemsa's stain. Peak parasitaemia occurred at 5–6 d post-infection, after which time parasite levels declined and remained at low but usually detectable levels for the remainder of experiments (Figure S5), as previously described [34]. Malaria-infected (day 12 of infection) and control mice were immunised intravenously with 500 μg of OVA (Sigma-Aldrich) or Eα-GFP [23], along with 50 ng LPS (from *Salmonella equi abortus*; Sigma-Aldrich).

### Preparation of bone marrow DCs.

DCs were prepared from BM as previously described [35]. Cell suspensions were obtained from femurs and tibias of female BALB/c mice. The BM cell concentration was adjusted to 5 × 10^5^ cells/ml and cultured in 6-well plates (Corning Costar) in complete RPMI (cRPMI: RPMI 1640 supplemented with L-glutamine [2 mM], penicillin [100 μg/ml], streptomycin [100 μg/ml] [all from Invitrogen], and 10% FCS [Labtech International]) containing 10% of culture supernatant from X63 myeloma cells transfected with mouse GM-CSF cDNA. Fresh medium was added to the cell cultures every 3 d. On day 6, DCs were harvested and cultured at the required concentration for each individual experimental procedure as described below. This technique generated a large number of CD11c+ DC largely free from granulocyte and monocyte contamination, as previously described [35]. DCs were antigen loaded for 6 h with 5 mg/ml OVA (Worthington Biochemical), 5 μg/ml OVA_323–339_ peptide (Sigma-Genosys), or 100 μg/ml Eα-GFP [23], and/or stimulated with 1 μg/ml LPS prior to use, as indicated in individual experiments. In imaging experiments, DCs were fluorescently labelled with Cell Tracker Orange (CMRA; Invitrogen) or 5,6-carboxy-succinimidyl-fluorescein-ester (CFSE; Invitrogen) immediately before use [36]. For in vivo imaging, 1 million DCs were immunised subcutaneously in the footpad of T cell recipient animals and the draining popliteal LN imaged 20–24 h later.

### Antigen-specific T cells.

DO11.10/scid LNs and spleens were homogenised and resulting cell suspensions washed twice and resuspended in RPMI. Cells were labelled with the fluorescent dye CFSE or Cell Tracker Red (CMTPX; Invitrogen) immediately before use [36]. Syngeneic BALB/c recipients received 3 × 10^6^ to 6×10^6^ antigen-specific cells.

For functional in vitro studies, OVA-specific T cells were mixed with HZ-treated or control BM DCs at a 1:1 ratio in 96-well tissue culture plates (Corning Costar). T cell proliferation was assessed after 72 h of culture and assessed by incorporation of [^3^H] thymidine (0.5 μCi/well) for the last 24 h of culture. To measure in vitro clustering of T cells with DC, cells were co-cultured in 12-well plates for 20 h and then fixed with 4% paraformaldehyde (Sigma-Aldrich; 20 min at 4 °C). Gly-Gly (0.06%; Sigma-Aldrich) was added and briefly incubated for 1 min to neutralise residual paraformaldehyde. Cells were then harvested and analysed by flow cytometry.

### Flow cytometry.

Aliquots of 1 × 10^6^ cells in 12 × 75 mm polystyrene tubes (BD Biosciences) were resuspended in 100 μl of FACS buffer (PBS, 2% FCS and 0.05% NaN_3_) containing Fc Block (2.4G2 hybridoma supernatant) as well as the appropriate combinations of the following antibodies: anti-CD4-PerCP (clone RM4–5), anti-CD11c-PE (clone HL3), anti-CD40-FITC (clone 3/23), anti-CD69-PE (clone H1.2F3), PE-hamster IgG isotype control and FITC-rat IgG2a, k isotype control (all BD Biosciences), biotinylated KJ1.26 antibody, or biotinylated-Y-Ae. Biotinylated antibodies were detected by incubation with fluorochrome-conjugated streptavidin (BD Biosciences). After washing, samples were analysed using a FACSCanto flow cytometer equipped with a 488-nm Argon laser and a 635-nm red diode laser (BD BioSciences) and analysed using FlowJo software (Tree Star).

### HZ preparation.

Synthetic HZ was produced using the method of Egan et al [22]. Briefly, hemin chloride (Sigma-Aldrich) was polymerised using 4.5 M sodium acetate at 60 °C and the product extensively washed with deionised water and filtered using 0.22-μm cellulose nitrate filtration units. Endotoxin-free buffers and solutions were used throughout. *Plasmodium* HZ was isolated from supernatants obtained from cultures of P. falciparum gametocytes, kindly provided by Lisa Ranford-Cartwright, Division of Infection and Immunity, University of Glasgow, UK. Supernatants were centrifuged for 20 min at 450*g*. The pellet was washed three times in 2% SLS and resuspended in 6 M guanadine HCl. Following five to seven washes in PBS, the pellet was resuspended in PBS and sonicated for 90 min using Soniprep 150 (Sanyo Scientific) at an amplitude of 5–8 μm. Again, endotoxin-free buffers were used throughout. Total haem content was determined as previously described [37] by depolymerising haem in 1 ml of 20 mM NaOH/2% SDS, incubating the suspension at room temperature for 2 h, and then reading the OD at 400 nm using UV visible spectrophotometer (Thermo Spectronic, Heλios). Prior to use, the HZ was sonicated to minimise aggregation and maintain the HZ in suspension. DCs were pulsed with 1–40 μM HZ, a range similar to that seen when DCs were cultured at 1:100 ratio with P. chabaudi–infected erythrocytes.

### In vitro and in vivo imaging.

For in vitro imaging, 5 × 10^4^ CMRA-labelled DC and 5 × 10^4^ CFSE-labelled DO11.10 T cells were co-cultured on an Ibidi μSlideVI (Thistle Scientific). Time-lapse images were acquired using an Axiovert S-100 Zeiss microscope using a ×63 oil immersion lens 20 h after mixing of cells. For the images in Figure 1, 40 μM HZ was added in RPMI at the initiation of imaging.

To image cellular interactions in LNs, the excised LNs were transferred into CO_2_-independent medium (Invitrogen) at room temperature. The LN was bound with veterinary glue (Vetbond, 3 M) onto a coverslip that was then adhered with grease to the bottom of the imaging chamber and continuously supplied with warmed (36.5 °C) and gassed (95% O_2_ and 5% CO_2_) RPMI 1640 before and throughout the period of microscopy. Excised LNs were imaged on the following system, as previously described [5,38]. The two-photon excitation source was a solid-state, tunable Titanium: sapphire laser system (5W Chameleon; Coherent Laser Group). The laser beam was routed into a multi-photon excitation laser scanning system (Radiance; Bio-Rad Laboratories). The objective lens used for all imaging investigations was the CFi-60 Fluo-W 40X/0.8 water-dipping objective lens (Nikon). The sample was illuminated with 780–830 nm light, and the emission spectrum was separated with a 550-nm dichroic mirror (Chroma Technologies). Each imaged volume consisted of between 11 to 18 planes 2.55 μm apart. Volumes were acquired every 18 to 38 s. Images were analysed using Volocity software (Improvision). Objects were tracked for at least eight time points and the mean velocity, displacement, and meandering index calculated for each. The interaction between DCs and T cells was measured by quantifying the colocalisation of green voxels with red to generate the colocalisation coefficient—a measure of the proportion of DC volume in contact with T cells.

### Statistical analysis.

Results are expressed as mean ± standard deviation. Significance was determined by Student's *t*-test using Minitab. A *p*-value of *p* ≤ 0.05 was considered significant.

## Supporting Information

Figure S1 Synthetic HZ Recapitulates the Effects of *Plasmodium* HZ on DCs BM DCs were treated with synthetic HZ (sHZ) or HZ isolated from P. falciparum gametocytes (Pf HZ) for 18 h. Following treatment, DCs were stimulated with 1 μg/ml LPS for a further 24 h before analysis of activation by flow cytometry. Results show the mean (± 1 s.d.) proportion of CD11c+ DCs expressing CD40.
(46 KB JPG)

Figure S2 Quantitation of DC–T Cell Interactions by Flow Cytometry CMRA-labelled DCs and CFSE-labelled DO11.10 T cells were co-cultured for 20 h with or without 5 mg/ml OVA as indicated. Supernatants were removed and the cells fixed with 4% paraformaldehyde at 4 °C for 20 min prior to analysis by flow cytometry. Individual populations of (a) CMRA+ DCs and (b) CFSE+ T events could be identified in mixed cultures (c). Following OVA-pulse of DCs (d), an increased proportion of events were CMRA+CFSE+ (upper-right quadrant), indicating colocalisation between DCs and T cells. Data are representative images from controls in three individual experiments.
(108 KB JPG)

Figure S3 Normal Antigen Processing and Presentation by DCs following Treatment with P. chabaudi–Infected Erythrocytes BM DCs were co-cultured for 18 h at 1:100 ratio with P. chabaudi–infected erythrocytes (pRBC), control erythrocytes (RBC), or alone. Cells were then pulsed with 100 μg Eα-GFP (filled bars) or remained unpulsed (empty bars). Antigen uptake (a) and presentation (b) by CD11c+ cells assessed 6 h later by flow cytometry. Results show the mean fluorescence ± 1 s.d. of triplicate samples and are representative of three similar experiments.
(93 KB JPG)

Figure S4 Comparison of Naïve T Cell Migration in Uninfected and P. chabaudi–Infected Animals BALB/c mice were infected with 10^6^ P. chabaudi AS-infected erythrocytes 12 d prior to the transfer of CFSE-labelled CD4+ T cells. Control animals remained uninfected. T cell motility in LN was imaged by multiphoton microscopy 20 h later. T cell behaviour was analysed using Volocity software and mean velocity calculated.
(51 KB JPG)

Figure S5 Kinetics of P. chabaudi Infection BALB/c mice were infected with 10^6^ P. chabaudi AS-infected erythrocytes and peripheral blood monitored for the appearance of parasites. The proportion of parasitised erythrocytes on Giemsa's-stained blood smears was monitored throughout the infection.
(50 KB JPG)

Video S1 Rapid Uptake of HZ by DCs BM DCs were imaged by time-lapse brightfield microscopy. HZ was added to the culture at the beginning of the sequence.
(4.2 MB MOV)

Video S2 Response of DCs to LPS Stimulation BM DCs were imaged by time-lapse brightfield microscopy before, during, and after the addition of LPS (added at 30 min).
(3.6 MB MOV)

Video S3 Response of HZ-Treated DCs to LPS Stimulation Cells were set up and imaged as in Video S2 except that DCs were treated with HZ prior imaging.
(3.9 MB MOV)

Video S4 Interaction of DCs with T Cells In Vitro CMRA-labelled, OVA-pulsed BM DCs (orange) were co-cultured with CFSE-labelled DO11.10 T cells (green) for 20 h. Cellular interactions were imaged by time-lapse brightfield and fluorescence microscopy.
(658 KB MOV)

Video S5 Interaction of HZ-Treated DCs with T Cells In Vitro Cells were set up and imaged as in Video S4 except that DCs were treated with HZ prior to pulse with OVA.
(8.0 MB MOV)

Video S6 Interaction of DCs with T Cells In Vivo CMTPX-labelled DO11.10 T cells (red) were transferred into BALB/c recipient animals which were subsequently immunised with CFSE-labelled BM DCs (green). Cellular interactions in the draining popliteal LN were imaged by multiphoton scanning microscopy 24 h later.
(140 KB MOV)

Video S7 Interaction of Antigen-Pulsed DCs with T Cells In Vivo Cells were transferred and imaged as in Video S6 except that DCs were pulsed with OVA_323–339_ prior to immunisation.
(2.2 MB MOV)

Video S8 Interaction of Antigen-Pulsed, HZ-Treated DCs with T Cells In Vivo Cells were transferred and imaged as in Video S6 except that DCs were treated with HZ and pulsed with OVA_323–339_ prior to immunisation.
(1.2 MB MOV)

Video S9 In Vivo Imaging of Naïve CD4+ T Cell Movement CFSE-labelled DO11.10 T cells were transferred into uninfected mice and the inguinal LN imaged by multiphoton scanning microscopy 20 h later.
(705 KB MOV)

Video S10 In Vivo Imaging of CD4+ T Cell Movement following Immunisation of Uninfected Animals CFSE-labelled DO11.10 T cells were transferred into uninfected mice. Animals were immunised with OVA/LPS intravenously and the inguinal LN imaged by multiphoton scanning microscopy 20 h later.
(1.4 MB MOV)

Video S11 In Vivo Imaging of Naïve CD4+ T Cell Movement following Immunisation of P. chabaudi–Infected Animals CFSE-labelled DO11.10 T cells were transferred into P. chabaudi-infected mice on day 12 of infection. Animals were immunised with OVA/LPS intravenously and the inguinal LN imaged by multiphoton scanning microscopy 20 h later.
(908 KB MOV)

## Abbreviations

BM: bone marrow
DC: dendritic cell
HZ: hemozoin
LN: lymph node
LPS: lipopolysaccharide
MHC: major histocompatibility complex
OVA: ovalbumin
Treg: regulatory T cell
